# Ultrasound Surveillance Offers a Safe and Effective Method for Venous Thromboembolism Prevention in Plastic Surgery Patients

**DOI:** 10.1007/s00266-020-01935-4

**Published:** 2020-08-31

**Authors:** Eric Swanson

**Affiliations:** Swanson Center, Leawood, KS 66211 USA

*Level of Evidence V* This journal requires that authors assign a level of evidence to each article. For a full description of these Evidence-Based Medicine ratings, please refer to the Table of Contents or the online Instructions to Authors www.springer.com/00266.

Five recent publications investigate venous thromboembolism (VTE) risk reduction [1–5]. Rochlin et al. [1] analyzed a large database and conclude that the length of hospital stay is a modifiable risk factor in microsurgical breast reconstruction patients. However, these authors found no significant difference in VTE rates over time comparing patients treated during 2007–2009 with women treated during 2013–2015, despite the fact that patients treated during the earlier time frame had significantly longer hospital stays (4.36 days versus 4.13 days) [1].

Pannucci, the senior author of this review [1], has long championed Caprini scores and individual risk stratification [6, 7]. Importantly, the length of hospital stay is not considered in a Caprini score [7, 8]. In fact, Caprini [8] believes that patients are just as sedentary at home after discharge as they were in the hospital, remarking, “these individuals spend most of the time in a recliner, which is not early ambulation but rather early angulation.” Although the 2011 Venous Thromboembolism Prevention Study [7] determined that length of stay correlates with VTE risk, the hospital stay subgroups were too small to be statistically reliable [9]. Surprisingly, the VTE risk for patients treated with and without chemoprophylaxis was the same, 1.2% [7].

Rochlin et al. [1] suggest that shortening the hospital stay may be helpful in reducing VTE risk. This is a difficult thesis to prove because patients who are discharged sooner are also likely to be healthier. Sicker patients typically have longer hospital stays, making it impossible to isolate length of stay from patient health. It would be unwise to start discharging patients too soon from the hospital under the mistaken assumption that the longer they stay in the hospital the more likely they are to develop a VTE.

Aimé et al. [2] report the findings of a survey of plastic surgeons regarding VTE risk reduction methods in aesthetic surgery. The authors reiterate the conventional wisdom endorsing Caprini scores and anticoagulation for patients deemed to be at high risk [2]. Three other studies published within the last year also support chemoprophylaxis [3–5].

Notably, four of the five studies do not discuss or reference ultrasound surveillance for VTE [1, 2, 4, 5], although 4.5% of surveyed plastic surgeons report using this modality for screening and management [2]. Today, ultrasound applications in plastic surgery are a subject of intense interest [10–14]. At the 2018 meeting of the American Society of Plastic Surgeons, ultrasound screening for deep venous thromboses was chosen as “Best of Hot Topics.” [12] The YouTube video [13] that accompanies the original 2015 publication [14] has now been viewed over 166,000 times, greatly exceeding the number of active board-certified plastic surgeons in the USA (7075) [15]. Evidently, this method has caught the interest of other specialties and healthcare providers.

Published guidelines are cited to support risk stratification and chemoprophylaxis [1–5]. Most of these guidelines, including the 2012 Task Force recommendations of the American Society of Plastic Surgeons (ASPS) [16], were published before ultrasound surveillance for VTE risk reduction was introduced [14]. Guidelines are meant to be updated when new information becomes available. About half of medical guidelines are obsolete in 6 years [17].

New evidence is now available regarding the frequency, timing, anatomical location of deep venous thromboses, and their response to treatment in a large number (*n* = 1000) of aesthetic surgery outpatients [10]. This information is helpful in several ways:

First, this information is available for plastic surgery outpatients, avoiding the issue of confounding types of surgery (often general or orthopedic surgery), diagnoses (particularly cancer), and types of anesthesia. General anesthesia is associated with a greater VTE risk than total intravenous anesthesia without paralysis [18].

Second, the patients were all screened with Doppler ultrasound, which is a reliable method to detect deep venous thromboses [10]. Most existing studies, many decades old, depend on physical examination, which is unreliable, or on outdated techniques, such as impedance plethysmography, venography, or radioactive iodine-labeled fibrinogen [10]. D-dimer assays are not sufficiently sensitive for detecting distal thromboses [19].

Third, this study provides new information regarding the timing of VTEs (not during surgery as expected), which veins are affected (primarily distal), and how long deep venous thromboses take to resolve with treatment (5 weeks on average) [10]. This information is vital when considering when to initiate anticoagulation and for what period of time. The average duration of anticoagulation reported by surveyed plastic surgeons is 6 days [2]. The evidence shows that even a 1-week course of enoxaparin is unlikely to be effective in preventing VTEs or in treating those that occur—the course is too early and ends too quickly to be effective [10]. Distal (calf) deep venous thromboses often produce no symptoms or leg swelling, making ultrasound essential for their detection [20].

If treatment recommendations are based on Caprini scores, their limitations must be recognized. Many plastic surgeons are unaware that Caprini based his scores on emotion, logic, experience, and intuition [21]. These considerations do not meet the bar for evidence-based medicine. Not surprisingly, Caprini scores do not correlate significantly with relative risk data obtained from the scientific literature [21]. Many of the scores under-estimate (e.g., immobilization and bed rest, 1 point) or over-estimate (e.g., serum homocysteine level, 3 points) relative risk [21]. Hospitalization, long periods of travel, and the type of anesthesia are omitted [8].

When analyzing survey data, it is difficult to determine whether traditional risk factors persist once age, the most important variable [10, 22], is considered. Although they are frequently cited as risk factors, body mass index, hormonal supplementation, and smoking history do not significantly affect VTE risk in plastic surgery outpatients [10]. Several parameters, including operating time, number of procedures, abdominoplasty, and age do significantly correlate with VTE risk [10]. However, only age persists as a significant factor when considering the effect of covariates using logistic regression [10]. Indeed, patients undergoing abdominoplasty and combined procedures, which take longer, also tend to be older. This finding is consistent with our understanding that deep venous thromboses are triggered by hypoxia in the venous valve sinuses [18, 22]. Older patients have stiffer valves [22].

Surprisingly, proponents of individual risk stratification do not always use this method when prescribing chemoprophylaxis [3, 4, 23]. Vasilakis et al. [3] prescribed enoxaparin to abdominal body contouring patients regardless of Caprini scores. In a subsequent study, Vasilakis et al. [4] prescribed rivaroxaban to all abdominoplasty and body lift patients. Similarly, Momeni et al. [23] treated all breast reconstruction patients with enoxaparin.

Regulatory issues cannot be ignored. The US Food and Drug Administration (FDA) approves enoxaparin for VTE prophylaxis only in high-risk general surgery and joint replacement patients [24]. Chemoprophylaxis in plastic surgery is not FDA-approved; prescribing anticoagulation for this purpose is off-label. By contrast, ultrasound devices are FDA-cleared [25] and anticoagulation of diagnosed deep venous thromboses is FDA-approved [25]. Remarkably, a method that is not approved by the FDA for plastic surgery patients is promoted as the standard of care while an FDA-approved method is dismissed as outside the standard of care [3].

Screening for deep venous thromboses has a highly rational basis. The diagnosis comes before treatment rather than the reverse, in accordance with time-honored medical practice. Alarmingly, 10% of symptomatic pulmonary embolisms present with sudden death [26]. Deep venous thromboses start in the distal veins, where the risk of pulmonary embolism is low (less than 5%) [20]. Without detection, thrombi may propagate. Once a deep venous thrombosis spreads to the proximal veins of the thigh, the risk of pulmonary embolism increases dramatically (50%) [20].

As a practical matter, compliance will always be a problem with regard to healthcare personnel taking the time to assess a Caprini score or with patients injecting themselves at home. Plastic surgeons have legitimate concerns regarding bleeding risk [10]. When prescribing enoxaparin, most surgeons start anticoagulation 6–8 h after surgery and continue injections for the duration of the patient’s hospitalization [10, 27]. Preoperative injections may increase the risk of bleeding [19].

One recent study reported 64 hematomas among 1128 (postoperatively) anticoagulated abdominoplasty patients (5.7%) [27]. Hematomas should not be considered an acceptable trade for a VTE [27], particularly if a VTE can be identified early in its development, when it is not dangerous, and managed with minimal disruption to the patient’s activities and recovery [10]. Hematomas are distressing to patients and surgeons. If offered the choice, patients prefer ultrasound screening to routine anticoagulation [10].

Oral anticoagulants such as rivaroxaban and apixaban are better tolerated by patients than enoxaparin injections but are not without risk [28]. Dini et al. [29] reported an alarming frequency of hematomas in abdominoplasty patients who were prescribed rivaroxaban after surgery. Like enoxaparin, oral anticoagulants are not FDA-approved for VTE prevention in plastic surgery patients [28]. In the USA, attorneys actively solicit patients who experience bleeding complications after taking rivaroxaban [28].

**Fig. 1:**
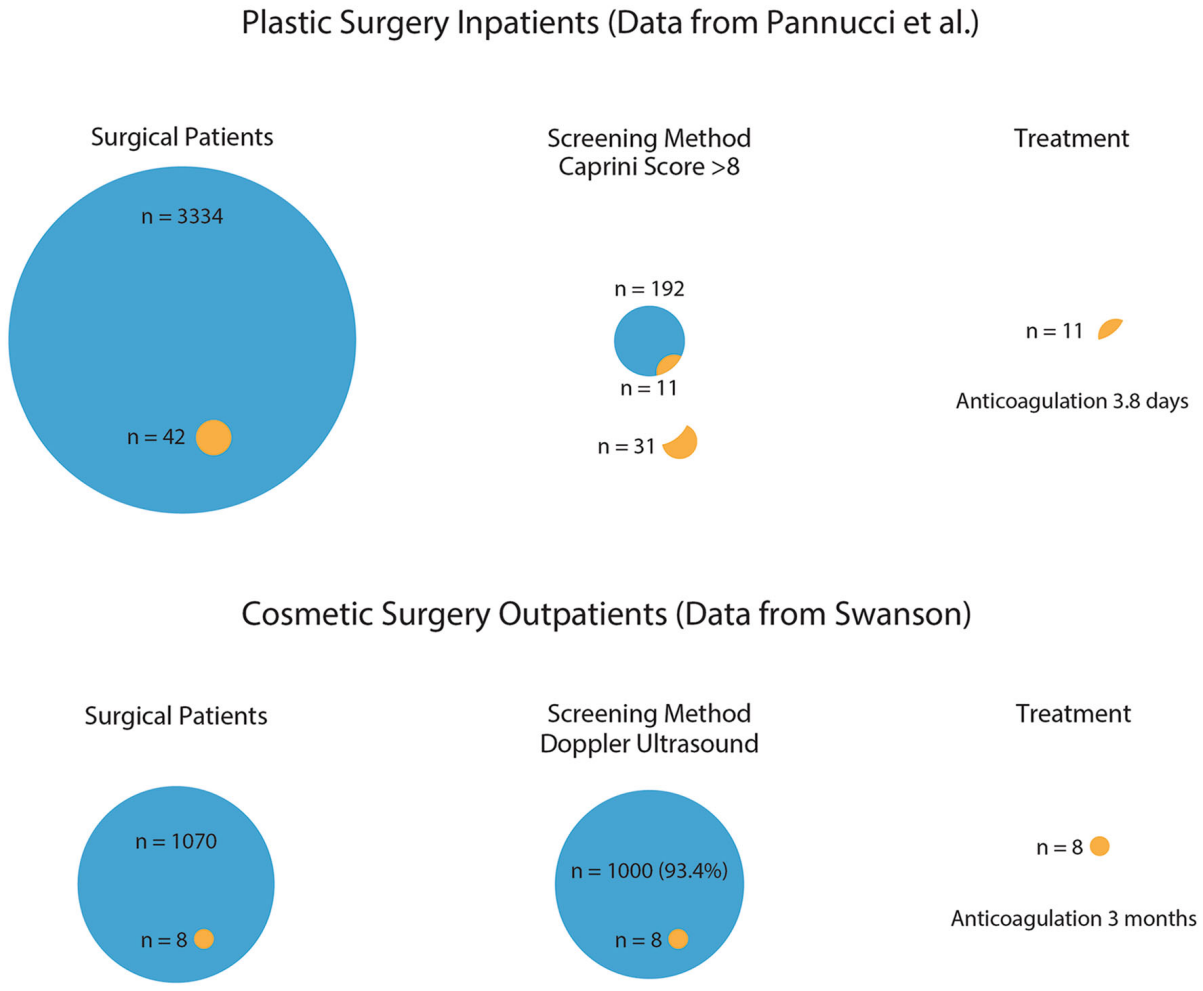
Risk stratification and chemoprophylaxis (above) is compared with Doppler ultrasound imaging and anticoagulation for affected individuals (below). Using the data from Pannucci et al. [7] obtained from plastic surgery inpatients, a Caprini score of > 8 identified 11 of 42 affected patients (26%). These patients were treated with enoxaparin for the duration of their hospitalization (mean, 3.8 days) [7]. By contrast, ultrasound imaging detected all deep venous thromboses presenting within 30 days of surgery [10]. Affected patients received a 3-month course of oral anticoagulation and were monitored with weekly ultrasound scans to ensure resolution (mean time to resolution, 5 weeks) [10]

Efforts to predict affected patients have frustrated investigators (Fig. 1). Lemaine et al. [30] reported that 96.6% of microsurgical breast reconstruction patients who were categorized as highest risk had no ultrasonic evidence of deep venous thromboses. Shaikh et al. [31] reported no VTEs among 36 patients with “super high” Caprini scores exceeding 10. Keyes et al. [32] concluded that Caprini scores were unhelpful because 67% of abdominoplasty patients who experienced VTEs had Caprini scores less than 6.

Even its proponents concede the limitations of Caprini scores, particularly in aesthetic surgery patients, and now encourage risk mitigation instead as the “dominant initial strategy.” [33] However, there is little evidence that such measures are clinically effective [10]. Aimé et al. [2] note that only 1 in 200 aesthetic surgery outpatients has a 2005 Caprini score of 7 or more. Consequently, 200 Caprini scores must be calculated to identify a single patient for whom anticoagulation is recommended.

Adopting ultrasound does require the purchase of new equipment and the services of a trained operator. The cost for three studies is about $200 for a practice that regularly uses ultrasound [20, 28]. Alarmingly, 6% of surveyed plastic surgeons have encountered a patient death from a VTE [2]. Any plastic surgeon who has experienced a patient suffer a catastrophic VTE will find that the cost of ultrasound surveillance is not a barrier. Doppler ultrasound is highly accurate and sensitive for the detection of deep venous thromboses (Fig. 1) [10]. The author does not charge patients for ultrasound screening. Patients are saved the expense of purchasing anticoagulants, except for those who develop a deep venous thrombosis.

Unlike in years past, today we have the technology. We can diagnose a deep venous thrombosis while it is small and distal, before it propagates, and initiate anticoagulation early and for a sustained period with follow-up scans to ensure resolution. Ultrasound screening provides early warning, a “canary in a coal mine,” so to speak [20]. It is far better to diagnose a deep venous thrombosis on ultrasound than on autopsy. Nevertheless, despite numerous publications and presentations supporting the efficacy and safety of ultrasound in VTE prevention [10–14, 19–21], this strategy is often overlooked [1, 2, 4, 5]. Scientifically, one can accept contradictory evidence or reject it, but not ignore it.

Ultrasound is a disruptive technology. Resistance to a change in practice is to be expected, but plastic surgeons generally welcome innovation [11–13]. Plastic surgeons frequently purchase new technologies (e.g., radiofrequency, lasers) that are much more expensive than an ultrasound device. Soon they find ultrasound an indispensable tool for many other office applications [11]. Plastic surgeons have an opportunity to take the lead in adopting this innovative technology. Surgeons may discard ineffective and time-consuming efforts to predict affected patients (with poor compliance even among its advocates) [3, 4, 23]. It is time to embrace ultrasound technology. This is the future direction of VTE prevention.
